# T‐cell epitope content comparison (EpiCC) of swine H1 influenza A virus hemagglutinin

**DOI:** 10.1111/irv.12513

**Published:** 2017-11-28

**Authors:** Andres H. Gutiérrez, Vicki J. Rapp‐Gabrielson, Frances E. Terry, Crystal L. Loving, Leonard Moise, William D. Martin, Anne S. De Groot

**Affiliations:** ^1^ Department of Cell and Molecular Biology Institute for Immunology and Informatics University of Rhode Island Providence RI USA; ^2^ Zoetis Inc. Kalamazoo MI USA; ^3^ EpiVax Inc. Providence RI USA; ^4^ Virus and Prion Diseases Research Unit NADC, USDA ARS Ames IA USA

**Keywords:** computational immunology, hemagglutinin, influenza A viruses, swine influenza H1 viruses, swine leukocyte antigen, T‐cell epitope content comparison, T‐cell epitope prediction, vaccine efficacy

## Abstract

**Background:**

Predicting vaccine efficacy against emerging pathogen strains is a significant problem in human and animal vaccine design. T‐cell epitope cross‐conservation may play an important role in cross‐strain vaccine efficacy. While influenza A virus (IAV) hemagglutination inhibition (HI) antibody titers are widely used to predict protective efficacy of 1 IAV vaccine against new strains, no similar correlate of protection has been identified for T‐cell epitopes.

**Objective:**

We developed a computational method (EpiCC) that facilitates pairwise comparison of protein sequences based on an immunological property—T‐cell epitope content—rather than sequence identity, and evaluated its ability to classify swine IAV strain relatedness to estimate cross‐protective potential of a vaccine strain for circulating viruses.

**Methods:**

T‐cell epitope relatedness scores were assessed for 23 IAV HA sequences representing the major H1 swine IAV phylo‐clusters circulating in North American swine and HA sequences in a commercial inactivated vaccine (FluSure XP ^®^). Scores were compared to experimental data from previous efficacy studies.

**Results:**

Higher EpiCC scores were associated with greater protection by the vaccine against strains for 23 field IAV strain vaccine comparisons. A threshold for EpiCC relatedness associated with full or partial protection *in the absence of cross‐reactive HI antibodies* was identified. EpiCC scores for field strains for which FluSure protective efficacy is not yet available were also calculated.

**Conclusion:**

EpiCC thresholds can be evaluated for predictive accuracy of protection in future efficacy studies. EpiCC may also complement HI cross‐reactivity and phylogeny for selection of influenza strains in vaccine development.

## 1 INTRODUCTION

Influenza A viruses (IAVs) cause a highly contagious respiratory disease in swine, a problem that has a significant impact on food production.^1^ Predicting whether existing vaccines will protect against newly emergent strains circulating in pig herds is a significant problem for pork producers. Hemagglutinin (HA) and neuraminidase (NA) are the 2 major surface glycoproteins that define IAV subtypes and play a key role in antigenicity, pathogenesis, host range, and protection in the context of vaccination. The segmented IAV genome allows for antigenic shift by reassortment of RNA segments from different viral strains infecting the same cell, generating novel viruses.^2^ Antigenic drift due to accumulation of mutations in HA and NA contributes to the remarkable diversity of IAVs^2^ and impedes the development of broadly effective IAV vaccines for both pigs and humans.

Currently, H1N1, H1N2, and H3N2 are the predominant IAV subtypes cocirculating in the North American swine population. These subtypes are further subdivided based on the genetic and antigenic properties of HA. For H1 viruses, 7 distinct genetic phylo‐clusters (α, β, γ, γ‐2, δ1, δ2, and pandemic (pdm09)) have been identified.^3^ The HA gene of α, β γ, and pdm09 cluster viruses is most similar to classical swine H1N1 (cH1N1).^3^ HA from human‐origin δ viruses can be differentiated in 2 subclusters, δ1 and δ2.^4^ Due to antigenic variability of HA, serological cross‐reactivity between H1 clusters similar to cH1N1 is variable, and there is very limited serologic cross‐reactivity between these viruses and the even more divergent δ cluster viruses.^3^, ^5^ Whether for swine IAV or for human IAV, methods for predicting whether a vaccine will protect against emerging IAVs, *when serological cross‐reactivity is absent*, are needed.

Most of the North American commercial vaccines against swine IAV contain inactivated viruses. The predominant antibody responses induced by these vaccines are to the HA protein.^1^, ^6^ Therefore, hemagglutination‐inhibiting (HI) antibody titers, in addition to sequence analysis of HAs, are used to evaluate the potential for 1 vaccine to protect against variant strains.^6^ However, full or partial protection in pigs can still be observed (based on reduction in lung lesions and viral titers) following vaccination with inactivated commercial vaccines, *despite the absence of cross‐reactive antibodies post‐vaccination*.^1^, ^6^, ^7^, ^8^, ^9^, ^10^, ^11^, ^12^

Protection in the absence of neutralizing antibodies is attributed to T cell–mediated responses to cross‐conserved T‐cell epitopes.^13^, ^14^ For example, in humans, immunity to cross‐conserved epitopes during the 2009 H1N1 IAV pandemic may have contributed to attenuation of morbidity in some age groups.^13^, ^15^ In mice, a DNA vaccine based on 8 HA T‐cell epitopes and 1 NA epitope that were conserved between seasonal and pdm09 strains lowered lung viral loads in HLA‐DR3 transgenic mice challenged with pdm09 in the absence of antibody responses.^16^ Other murine studies have shown that cross‐protection induced by conserved antigens does not provide complete protection against infection, but reduces mortality, morbidity, virus replication, and viral shedding.^17^, ^18^ Protection in these instances is believed to be due to the recognition of conserved, linear T‐cell epitopes presented by class I or class II major histocompatibility complex (MHC) molecules to cytotoxic T lymphocytes (CTL, CD8) and T‐helper (Th, CD4) lymphocytes.^19^

As T‐cell epitopes that are similar in vaccine and challenge strains may be responsible for protection in the absence of cross‐reactive antibodies, we developed a method for T‐cell epitope content comparison (EpiCC) that assesses the relatedness of class I and II epitopes across antigens and predicts potential vaccine efficacy based on a relatedness threshold. Using this method, we evaluated whether T‐cell epitope relatedness could explain protection of pigs that were vaccinated with a commercial inactivated vaccine and challenged with heterologous H1N1 IAV when cross‐reactive antibodies were absent.

EpiCC differs from strict sequence‐based methods for comparing vaccine and outbreak strains. It uses PigMatrix, an algorithm that predicts class I and II T‐cell epitopes specific to swine MHC (Swine Leukocyte Antigen, SLA) alleles^20^ while also analyzing the TCR‐facing residues of T‐cell epitopes, to predict and assess epitope similarities between input pathogen protein sequences. PigMatrix and associated tools that comprise the iVAX vaccine design toolkit were previously validated in retrospective and prospective studies of SLA‐restricted influenza epitopes.^21^, ^22^ Here, we used EpiCC to determine the T‐cell epitope relatedness of HA proteins from 23 swine IAV strains representing the major H1 phylo‐clusters circulating in the North American swine population. As the internal genes in North American swine influenza have been highly conserved between strains since 1998 (due to the emergence of the triple‐reassortant internal gene [TRIG] cassette), we assumed minimal T‐cell epitope differences in those antigens and focused our analysis instead on the critical and most variable swine IAV antigen, HA.^3^, ^23^, ^24^ Comparing the results of previously performed vaccine efficacy studies with IAV EpiCC scores, we were able to identify a level of T‐cell epitope relatedness for a γ‐cluster H1N1 vaccine virus that could be associated with full or partial vaccine efficacy for this set of 23 IAV strains.

## 2 METHODS

### 2.1 Sequences

Hemagglutinin sequences from 23 H1 IAV strains were included in the analysis (Table 1). Sequences were either obtained from GenBank or provided by Zoetis. Twenty of these HA sequences were from swine H1 viruses representing the α, β, γ, γ‐2, δ1, δ2, and pdm09 phylo‐clusters, and 3 of the sequences were from strains (belonging to the γ, δ1, and δ2 H1 phylo‐clusters) included in FluSure XP (FS; Zoetis Inc., Florham Park, NJ, USA). Phylogenetic analysis was performed the using MEGA7.^25^ All 23 HA amino acid sequences were aligned with MUSCLE, and an evolutionary tree was defined using the maximum‐likelihood method with 500 bootstrap replicates.

**Table 1:** Hemagglutinin sequence information for swine H1 influenza A viruses

Virus name^a^	Virus H1 cluster	Label^b^	GenBank accession or source^c^
A/swine/Iowa/15/1930 (H1N1)	Classical	IA30 cH1	EU139823
A/swine/Illinois/02450/2008 (H1N1)	α	IL08 H1α	CY099052
A/swine/South Dakota/A01823598/2015 (H1N2)	α	SD15 H1α	KT356682
A/swine/St‐Hyacinthe/106/1991 (H1N1)	α	SH91 H1α	U11857
A/swine/Iowa/40766/1992 (H1N1)	α	IA92 H1α	KP788773
A/swine/Minnesota/00040/2002 (H1N1)	β	MN02 H1β	Zoetis
A/swine/Iowa/00239/2004 (H1N1)	β	IA04 H1β	KM198690
**A/swine/Iowa/110600/2000 (H1N1)**	γ	IA00 H1γ FS	Zoetis
A/swine/Minnesota/PAH618/2011 (H1N1)	γ	MN11 H1γ	Zoetis
A/swine/Ohio/02973/2010 (H1N1)	γ	OH10 H1γ	Zoetis
A/swine/Iowa/A01940123/2015 (H1N1)	γ	IA15 H1γ	KT699044
A/swine/Minnesota/A01940015/2015 (H1N1)	γ	MN15 H1γ	KT595733
A/swine/Iowa/A01410129/2012 (H1N1)	γ2	IA12 H1γ‐2	KJ397936
A/California/04/2009 (H1N1)	H1N1pdm09	CA09 H1pdm	GQ117044
A/swine/Oklahoma/0726H/2008 (H1N2)	δ1	OK08 H1δ1 FS	Zoetis
A/swine/Ontario/55383/04 (H1N2)	δ1	ON04 H1δ1	DQ280212
A/swine/Illinois/PAH710/2011 (H1N2)	δ1	IL11 H1δ1	Zoetis
**A/swine/South Dakota/A01823304/2015 (H1N2)**	δ1	SD15 H1δ1	KT277819
A/swine/Oklahoma/A01566774/2014 (H1N2)	δ1	OK14 H1δ1	KP270784
A/swine/Minnesota/A01823864/2015 (H1N2)	δ1	MN15a H1δ1	KT699050
A/swine/Iowa/A01823426/2015 (H1N2)	δ1	IA15 H1δ1	KT356694
A/swine/Minnesota/A01940042/2015 (H1N2)	δ1	MN15b H1δ1	KT733589
**A/swine/North Carolina/031/2005 (H1N1)**	δ2	NC05 H1δ2 FS	Zoetis
A/swine/NC/00573/2005 (H1N1)	δ2	NC05 H1δ2	FJ638306

a FS viruses are shown in bold font.

b FS viruses have “FS” at the end of their labels.

c Sequences marked “Zoetis” were provided by Zoetis and are considered proprietary.

### 2.2 MHC binding prediction

The HA amino acid sequences of the 23 IAV strains were screened using PigMatrix.^20^ PigMatrix parses sequences into 9‐mers and assesses the binding potential of each 9‐mer *i* to SLA class I and II alleles. For each individual allele *a* in a set of MHC alleles ***A***, PigMatrix raw scores *r* are normalized to Z‐scores using the average μ and the standard deviation σ of scores calculated for 100 000 random 9‐mers as previously described for EpiMatrix (a human T‐cell epitope prediction tool).^26^

$$Z{\left(i\right)}_{a}=\frac{\left(r-\mu \right)}{\sigma }$$

In this normalized set of scores for each SLA allele, 9‐mers with Z‐scores above 1.64 comprise the top 5% of sequences with significant SLA binding potential. Increasing Z‐scores correlate with higher MHC binding probability. The same thresholds for defining low, medium, and high probability MHC binders are applied as have been previously used in EpiMatrix studies.^26^

The distribution of SLA alleles among pig herds in the United States is unknown. Binding was therefore predicted to a set of SLA class I and II alleles that were frequently expressed in a cohort tested in a previous study (SLA‐I: SLA‐1*0801, 1*1201, 1*1301, 2*0501, 2*1201, 3*0501, 3*0601, and 3*0701; SLA‐II: SLA‐DRB1*0201, 0402, 0602, 0701, and 1001).^22^

As data are lacking on breadth of coverage for SLA (the ability for selected SLA alleles to cover an outbred population of pigs), the set of sequences was also evaluated using HLA alleles for which the breadth of coverage is known.^27^, ^28^ We quantified the HLA‐restricted T‐cell epitopes that could be identified in these IAV sequences and compared to the epitopes uncovered using the set of SLA selected for this study. The breadth of coverage comparison was performed using the following HLA class I and class II supertype alleles: HLA‐I: A*0101, A*0201, A*0301, A*2402, B*0702, and B*4403; and HLA‐II: DRB1*0101, 0301, 0401, 0701, 1101, 1301, and 1501.^29^

### 2.3 T‐cell epitope content comparison

EpiCC assesses the relatedness of T‐cell epitopes contained in a protein sequence of a strain *s* and those contained in a protein sequence of a vaccine strain *v* based on a comparison of the epitope sequences and their PigMatrix SLA binding score, using a set of MHC alleles *A*. For any comparison, T‐cell epitopes can be either shared (conserved) between sequences, or unique to the strain, or unique to the vaccine. Thus, the EpiCC score for the comparison between *s* and *v* (EpiCC score or T‐cell epitope‐based relatedness) is based on the PigMatrix scores of shared and unique epitopes (Fig. S1).

Intuitively, the epitope content of a protein depends on its epitope density. So, if a “high–epitope density” protein is compared to a highly similar protein and many of their epitopes are conserved or shared between the 2 strains, the scores of shared epitopes will be high; consequently, the score of the comparison of their epitope content (EpiCC score) will also be high. As PigMatrix binding probabilities of *s* and *v* are considered for the calculation, the EpiCC score will be even higher if the shared 9‐mer epitopes have high predicted binding probabilities to the alleles in the set *A*.

We hypothesized that if epitopes in a vaccine closely match the epitopes in the challenge strain, and vaccine‐ and strain‐unique epitopes are rare, the memory T cells induced by the vaccine are likely to recognize the epitopes in the proteins of the challenge strain. The model assumes (i) that there is no prior T‐cell memory to the epitopes (a naïve immune system); (ii) that vaccination does not induce memory T cells to epitopes that are unique to the challenge strain; and (iii) that the efficacy of the vaccine might be adversely affected by the presence of many vaccine‐unique epitopes.^30^ Consequently, in our calculation, the EpiCC score of 2 sequences is improved by the presence of shared epitopes but decreases with increasing numbers of strain‐ and vaccine‐unique epitopes. The impact of “unique” epitopes could be adjusted in future comparisons.

The first step in calculating the EpiCC score is to obtain T‐cell epitope predictions for *s* and *v* using PigMatrix. Each 9‐mer *i∈s* is compared to a corresponding 9‐mer *j∈v*. The pairs of 9‐mers *i*,*j* are determined from a local alignment of *s* and *v* sequences using the Smith‐Waterman algorithm from EMBOSS.^31^ For *i*,*j* where one of the 9‐mers has a gap in position 1, that 9‐mer is considered “nonexistent,” that is, excluded from comparison.

For each of the pairs *i,j* and for each allele *a∈A*, the score of a shared T‐cell epitope *S(i,j)a* is computed only for epitopes that are cross‐conserved (ie, *i*,*j* with identical residues that face the TCR and predicted to bind to allele *a*). We reasoned that epitopes with identical TCR‐facing residues (TCRf), which are also predicted to bind to the same MHC allele, are more likely to induce cross‐reactive memory T cells. This is a simple assumption because a TCR can recognize peptides with different TCRf,^32^ but it is a conservative initial approach to define potential cross‐reactive epitopes.^33^ For class I T‐cell epitope comparison, we assumed that *i* and *j* are cross‐conserved, and potentially cross‐reactive, if they have identical residues in positions 4, 5, 6, 7, and 8 and are predicted to bind to *a*, regardless of differences on their MHC‐facing amino acids. For class II, amino acids in positions 2, 3, 5, 7, and 8 were considered TCRf. Positions were selected based on published analysis of peptide‐MHC‐TCR crystal structures.^34^ *S(i,j)a* is calculated using predicted binding probabilities as follows:

$$S{\left(i,j\right)}_{a}=p{\left(i\right)}_{a}\cdot p{\left(j\right)}_{a}$$

where *p* is the cumulative probability in the normal distribution for the Z‐score. As the binding of *i* and *j* to allele *a* is independent, *S(i,j)a*, the probability of them both occurring, is the product of the probabilities of each occurring (ie, joint probability).

The score of a unique epitope *U(i,j)a* is determined for non‐cross‐conserved epitopes based on binding probabilities according to these criteria:

1. Score of a strain‐unique epitope: $if\;\;Z{\left(i\right)}_{a}>1.64\to U{\left(i,j\right)}_{a}=p{\left(i\right)}_{a}$
2. Score of a vaccine‐unique epitope: $if\;\;Z{\left(j\right)}_{a}>1.64\to U{\left(i,j\right)}_{a}=p{\left(j\right)}_{a}$

Note that for any given *i*,*j*, predicted epitopes cannot be both shared and unique for allele *a*, but they can be both strain‐unique and vaccine‐unique if *i* and *j* are predicted to bind allele *a*, and their TCRf are distinct. For *i,j* where both 9‐mers are not predicted to bind allele *a*,* S(i,j)a* and *U(i,j)a* are undetermined.

As the alleles in *A* are distinct, they are treated independently; hence, the score of shared epitopes for *i*,*j* over the full set of alleles can be calculated as a joint probability (ie, product of the shared binding probabilities for individual alleles). However, given that the score of a shared epitope is calculated only for *i*,*j* where both 9‐mers are predicted to be binders, the joint probability over multiple alleles underweights shared promiscuous epitopes. For this reason, we computed the sum of the probabilities instead.

For the calculation of the EpiCC score, we assume that binding of each 9‐mer epitope is mutually exclusive and uniform. Thus, *E* is the sum of shared and unique epitope scores of each *i*,*j* normalized by the total number of compared pairs *p* to account for variable epitope densities, and by the number of MHC alleles in *A* allowing for comparison of values of *E* determined using different numbers of MHC alleles. Formally, the EpiCC score for sequences from a vaccine and strain is computed as:

$$E{\left(s,v\right)}_{A}=\frac{1}{\left|p\right|\cdot \left|A\right|}\sum_{i\epsilon s;j\epsilon v}\sum_{a\epsilon A}S{\left(i,j\right)}_{a}-U{\left(i,j\right)}_{a}$$

The sum of class I and II *E(s,v)*_*A*_ is the total epitope‐based relatedness score for *s* and *v*. Note that *U(i,j)a* functions as a penalty; therefore, if $U{\left(i,j\right)}_{a}>S{\left(i,j\right)}_{a},\;E{\left(s,v\right)}_{A}$ is negative.

Comparison of the predicted epitope content of any sequence to itself defines the sequence's baseline EpiCC score (*E*(*s*,*s*)_*A*_; Fig. S1) and it represents the predicted epitope density of the sequence and the binding probabilities of its epitopes. It follows that the maximum value of any comparison between a vaccine strain and a challenge strain *E*(*s*,*v*)_*A*_ can only be less than or equal to *E*(*v*,*v*)_*A*_ or *E*(*s*,*s*)_*A*_. For vaccines with low T‐cell epitope content, the baseline EpiCC score *E*(*v*,*v*)_*A*_ will be low, and the comparison score *E*(*s*,*v*)_*A*_ will be also low, even if *s* and *v* epitopes are highly similar. Thus, low *E*(*s*,*v*)_*A*_ can be due either to low T‐cell epitope content of 1 or both sequences and/or to low epitope relatedness between the strains.

### 2.4 HA baseline EpiCC score comparison

We calculated the baseline EpiCC score of the HA sequence of each viral strain (*E*(*s*,*s*)_*A*_). So as to evaluate whether the selection of specific sets of MHC alleles had an effect on the baseline EpiCC scores, *E*(*s*,*s*)_*A*_ was calculated using the epitope content predicted with 4 different sets of MHC alleles. Specifically, we compared EpiCC scores calculated with swine allele sets SLA‐I and SLA‐II to EpiCC scores calculated with human allele sets HLA‐I and HLA‐II.

### 2.5 Comparison of HA T‐cell epitope content between field and vaccine viruses

To test whether we could determine an EpiCC score that defined full or partial protection, we compared the epitope content (predicted using SLA alleles) of each HA to that of FS vaccine viruses (*E*(*s*,*v*)_*A*_). Shared and unique class I, class II and total EpiCC scores were determined. We also explored the relationships between protein identity and EpiCC scores using regressions. To represent the lower end of the sequence identity spectrum, we included HA sequences from A/Missouri/2124514/2006 (H2N3), A/Guangxi/592/2011 (H5N1), A/swine/Mexico/GtoDMZC02/2014 (H5N2), A/swine/North Carolina/A01442548/2012 (H3N2), and A/swine/Missouri/A01727926/2015 (H4N6) viruses (GenBank accessions EU258939, KM027999, KU141372, KC445235, and KU641621, respectively); their HA amino acid sequences had identities between 41.1% and 64.61% when compared to the sequence of HA from FS viruses. A random sequence that had the same number of amino acids as the average HA sequence in this data set and the average (natural) amino acid frequencies of proteins in the Swiss‐Prot database was also included in the comparison.

### 2.6 Relationship between EpiCC scores and vaccine efficacy

The goal of this analysis was to determine whether a certain level of T‐cell epitope relatedness of HA could be associated with protection afforded by vaccination in the absence of cross‐reactive antibodies in pre‐challenge sera, assuming minimal variation in T‐cell epitope content among internal proteins. We therefore evaluated whether the experimental outcomes of six previously performed FS H1N1γ vaccination and H1N1 challenge studies could be predicted using a defined EpiCC score threshold. For this analysis, FS was considered to be protective if it significantly reduced the percentage of lung lesions and viral titers in nasal swabs (ie, nasal shedding) and/or in the lung or lung lavage. The EpiCC score threshold associated with protection was defined as the lowest EpiCC score for the comparison between the FS H1γ vaccine virus and challenge viruses, where studies demonstrated that the inactivated vaccine was protective. EpiCC scoring was performed independently and prior to obtaining information about the outcomes of the vaccination and challenge studies.

### 2.7 Statistical analysis

Wilcoxon matched‐pairs signed rank test was used to compare baseline EpiCC score of HA sequences defined using different sets of MHC alleles (eg, *E*(*s*,*s*)_*SLA‐I*_ vs *E*(*s*,*s*)_*SLA‐II*_; *E*(*s*,*s*)_*SLA‐I*_ vs *E*(*s*,*s*)_*HLA‐I*_). The same test was applied to evaluate differences between SLA class I and II EpiCC scores for each vaccine virus. Correlation between class I and II baseline EpiCC scores was determined using the nonparametric Spearman correlation coefficient (ρ). Correlation of class I and II EpiCC scores was evaluated using the same test. We used Pearson correlation (r) to evaluate the relationship between sequence identity and EpiCC scores. *P*‐values (*p*) less than .05 were deemed significant. Analyses were performed using R 3.3.1 (Foundation for Statistical Computing, Vienna, Austria).

## 3 RESULTS

### 3.1 Baseline EpiCC scores for *E*(*s*,*s*)_*A*_

**Figure 1:**
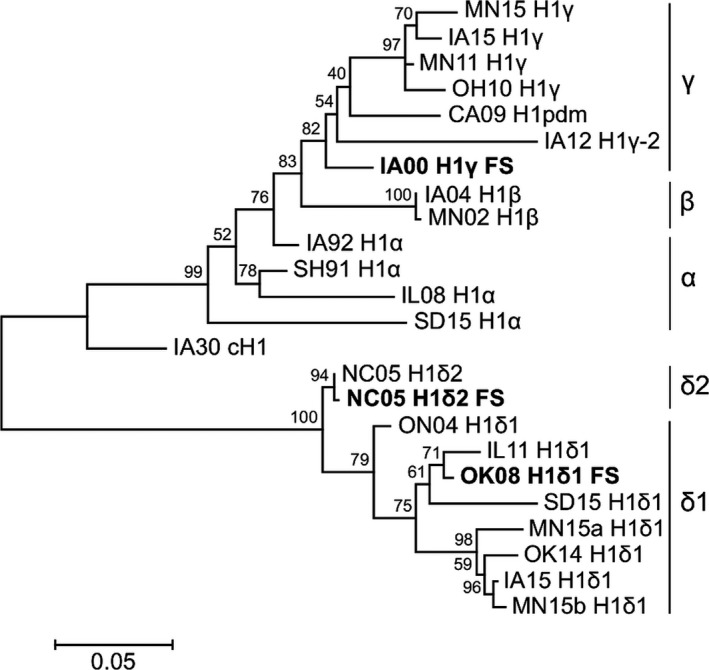
Phylogenetic tree for the Hemagglutinin (HA) amino acid sequences of influenza A field and FluSure vaccine (FS) viruses representing H1 phylo‐cluster in the North American swine. Bootstrap test results are shown next to the branches

HA amino acid sequences from different swine IAV phylo‐clusters (Figure 1) representing a range of sequence identities (from high to low) were analyzed. Although inactivated whole‐virus vaccines for influenza contain many antigens, the critical protective antigen (and the most variable) is considered to be HA. Thus, for this analysis, we calculated EpiCC for HA and did not include internal proteins in the calculations. Across the 23 H1 viruses, baseline HA SLA class I EpiCC scores, *E*(*s*,*s*)_*SLA‐I*_, were significantly lower (*P* < .001) and less variable (0.049 (0.001); mean (standard deviation)) than class II EpiCC scores (*E*(*s*,*s*)_*SLA‐II*_, 0.068 (0.004)) (Fig. S2A), and they were not significantly correlated (ρ = 0.18, *P* = .19). HA proteins from recently reported H1δ1 cluster viruses had the highest class II and total baseline EpiCC scores.

### 3.2 SLA vs HLA

To evaluate the effect of allele specificity and the breadth of coverage of the set of SLA alleles, we compared the baseline EpiCC scores predicted using SLA alleles to that predicted using human supertype HLA alleles. The baseline class I HLA allele‐defined EpiCC scores (*E*(*s*,*s*)_*HLA‐I*_, 0.063 (0.002)) were significantly higher (*P* < .001) than those predicted using SLA alleles (*E*(*s*,*s*)_*SLA‐I*_ (0.049 (0.001); Fig. S2B). Baseline HLA class II EpiCC scores (*E*(*s*,*s*)_*HLA‐II*_ (0.065 (0.004)) were significantly lower (*P* < .05) than those predicted using SLA class II alleles (*E*(*s*,*s*)_*SLA‐II*_ (0.068 (0.004); Fig. S2C). The relevance of this finding is unknown. The lower baseline scores observed for SLA class I alleles as compared to HLA class I alleles may indicate that the set of SLA‐I alleles selected for this study was not as broad in terms of population coverage of swine, and might not capture all the T‐cell epitope differences between strains. Alternatively, the HA sequences of the analyzed IAV may contain fewer epitopes that bind to SLA class I alleles. This could also be true for human class I epitope content in IAV, as the IAV strains had lower (*P* = .04) HLA class I baseline EpiCC scores compared to those of HLA class II using supertype HLA alleles (Fig. S2D). The number of reported class I epitopes defined for IAV in general (based on published data in the Immune Epitope Database^35^) also seems to be lower than the number of epitopes defined in IAV that are restricted by class II; thus, the differences described here are consistent with previous observations and may be relevant to pathogenesis.

### 3.3 Comparison of T‐cell epitope content between field and vaccine viruses

**Figure 2:**
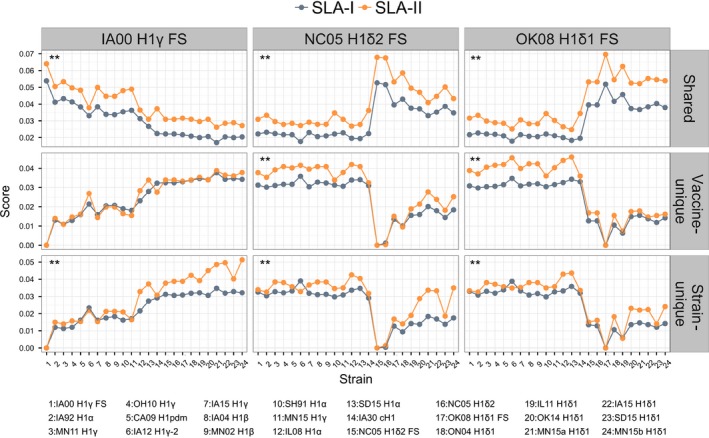
Comparison of scores of shared and unique epitopes across strains. Scores of shared, vaccine‐unique, and strain‐unique swine leukocyte antigen class I and II epitopes were determined for the comparison of hemagglutinin (HA) sequences from vaccine viruses and field (challenge) strains. Note that y‐axes show different scales. Solid connecting lines are included only for visualization purposes. P‐values of comparisons were calculated using 1‐tailed Wilcoxon matched‐pairs signed rank test (**P < .001). HA vaccine sequences had higher scores for shared epitopes with strains belonging to the same H1 cluster or the same HA lineage. In general, scores of class II shared and unique epitopes were significantly higher than those of class I. Viruses are sorted by nucleotide identity relative to H1γ FS. Strain numbers on the x‐axis are described in detail in the legend below

We then compared the SLA class I and II epitope content predicted for HA of each field virus to that of the vaccine viruses, *E*(*s*,*v*). Intuitively, HA proteins from similar strains will have similar epitope content. Thus, HA sequences from viruses within the same H1 cluster or in a cluster of the same HA lineage (cH1N1 or human seasonal) had higher scores for class I and II shared epitopes and lower scores for unique epitopes than viruses in clusters from a different HA lineage (Figure 2). It is noteworthy that there were shared epitopes in all comparisons, even when comparing viruses from different HA lineages.

Scores of shared, strain–unique, and vaccine‐unique SLA class II epitopes were significantly higher than those for SLA class I (*P* < .001 for the 3 vaccine viruses), except for scores of H1γ FS vaccine‐unique epitopes (*P* = .05). Likewise, using HLA supertype alleles, scores of class II shared epitopes were also significantly higher than those for class I (*P* < .01; Fig. S3). Class II scores of unique epitopes were also higher for OK08 H1δ1 FS (vaccine‐unique) and NC05 H1δ2 FS (strain‐unique). As mentioned above, although the population coverage of the set of SLA class I may be limited, the fact that the same lack of class I epitopes was observed for HLA supertype alleles suggests that the analyzed HA sequences had lower class I epitope content than class II content.

**Figure 3:**
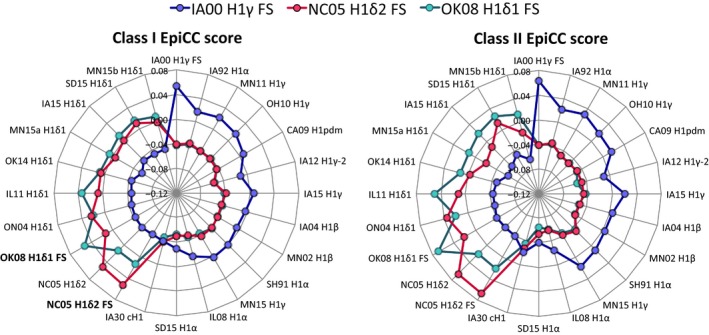
EpiCC score comparisons between hemagglutinin (HA) sequences of FluSure vaccine (FS) viruses and field viruses. Each axis corresponds to the HA sequence of 1 virus. EpiCC score = Scores of shared epitopes ‐ Scores of strain‐ and vaccine‐unique epitopes. HA sequences in the same cluster had the highest EpiCC scores. For each vaccine virus, class I and II EpiCC scores were significantly different from each other (P < .05). Note that for comparisons where the score for unique epitopes was greater than the score for shared epitopes, EpiCC scores were below zero

We used radar plots to facilitate the comparison of EpiCC scores between the vaccine and challenge strains. The EpiCC scores are displayed on these plots as a distance along a radiating line that also provides information on the relative sequence identity of the individual strains in the comparisons. In Figure 3, each axis corresponds to 1 virus HA sequence. The HA sequences are sorted clockwise by nucleotide identity, relative to the FS vaccine strain (IA00 H1γ FS virus). These radar plot figures highlight differences between EpiCC scores (distance) and sequence identity (see IA12 H1γ‐2 and IA15 H1γ, for example). The highest EpiCC score for each plot is given by *E*(*v*,*v*)_*A*_, that is, vaccine compared to itself.

**Figure 4:**
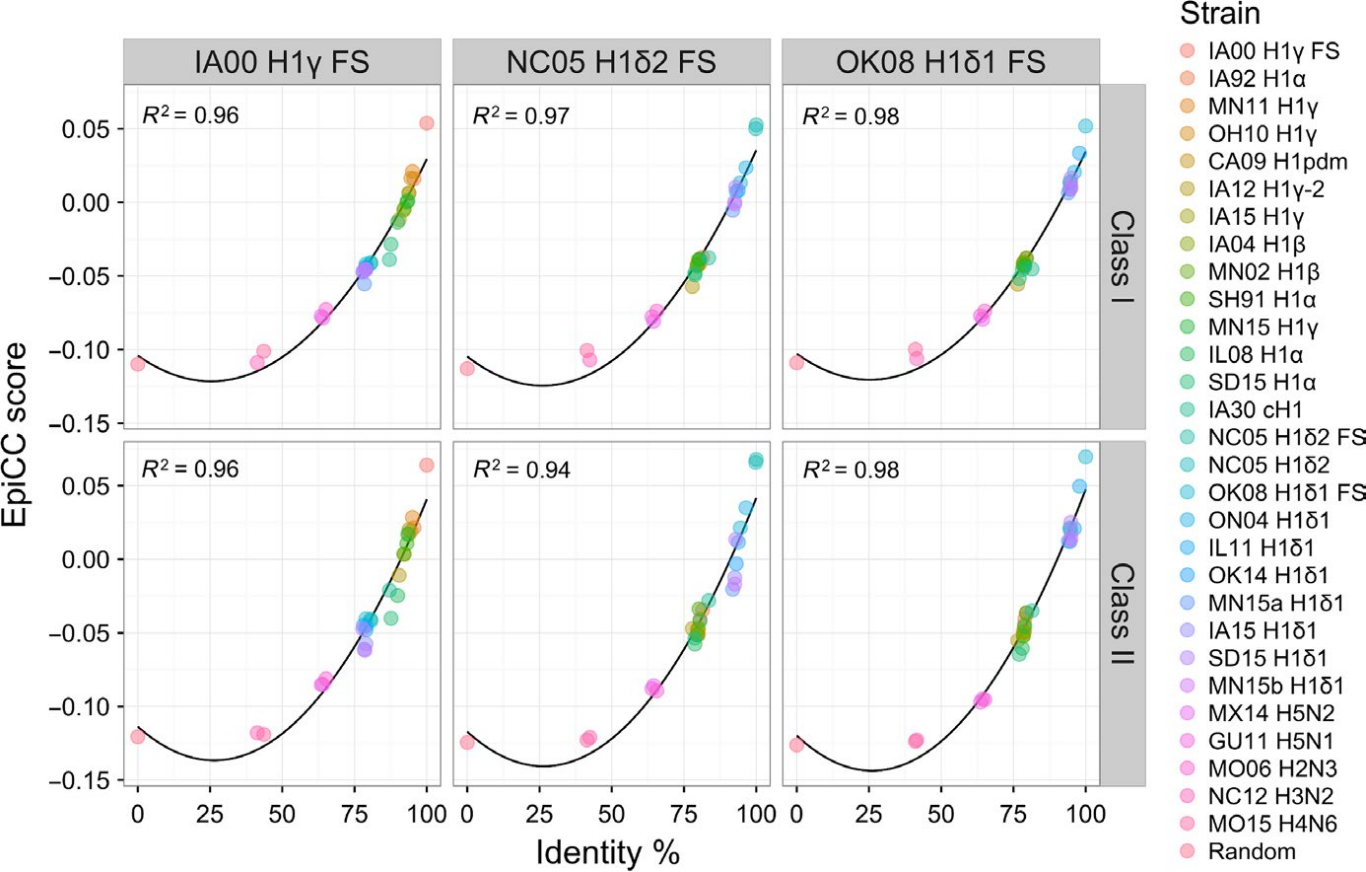
Relationship between EpiCC scores and hemagglutinin (HA) amino acid identity. The second‐order polynomial relationship between class I (top) and II (bottom) EpiCC scores and amino acid identity for each FS virus is shown. R_2_ values of regression models are shown. H2N3, H5N1, H5N2, H3N2, H4N6, and a random sequence were included in this analysis to represent the lower end of the identity range. Interestingly, there were instances where viruses had low EpiCC scores despite high identity (eg, class II epitope content of IA00 H1γ FS compared to SD15 H1α)

We also found that the relationship between identity and EpiCC scores was nonlinear second‐order polynomial (Figure 4; *R* ^2^=0.94‐0.98). Unlike identity, EpiCC scores reflect important differences in amino acids contained in predicted T‐cell epitopes and do not attribute value to amino acids that are not found in epitopes. For example, there are 35 amino acids that differ between the vaccine strain IA00 H1γ FS and the field virus CA09 H1pdm, but only 16 of these amino acids were involved in putative SLA binders and had an effect on the class II EpiCC score. Furthermore, only 4 of the residues were contained in 9‐mers that were predicted to bind to 3 or more class II SLA alleles. Thus, EpiCC score may more accurately reflect relevant immunological identity between sequences. EpiCC scores for sequences that had approximately 40% identity were no different from EpiCC scores for a random amino acid sequence of similar length. Similar results were observed for correlation with HA nucleotide sequence identity (Fig. S4).

### 3.4 Relationship between EpiCC scores and vaccine efficacy

Having calculated the EpiCC scores for 23 IAV strains, we then used the results of previously reported vaccine efficacy studies for the FS H1N1 γ‐cluster vaccine strain (IA00 H1γ FS) against heterologous viruses representing α, β, γ, or H1pdm clusters (Table 2) to define a threshold of HA T‐cell epitope relatedness between vaccine and challenge H1N1 strains that could be associated with protection. This information was not provided prior to conducting the EpiCC scoring. The primary measure for assessing vaccine efficacy in these studies was reduction in lung lesions; the reductions in viral nasal shedding and/or virus titers in the lung or lavage fluid at necropsy were considered to be secondary outcomes. The inactivated vaccine was considered protective in our analysis if there was a reduction in macroscopic pneumonia as well as reduction in virus titers in nasal swabs and/or in lung specimens collected at necropsy. If the vaccine significantly reduced virus titers, but not lung lesions, it was considered partially protective.

**Table 2:** FluSure XP^®^ vaccination and H1N1 challenge studies

Heterologous challenge	Measurement of protection^a^			HI GMT to challenge(vaccine)^b^virus	Outcome^c^	Ref.	Total EpiCC score(×10^2^)
H1N1 Virus	Percentage of macroscopic pneumonia	Virus titers in nasal swabs	Virus titers in lungs				
MN02 H1β	Reduced	Reduced	Not available	80 (381)	Protection	7	5.38
IA92 H1α	Significantly reduced	Significantly reduced	Not available	≤20 (320)	Protection	9	1.61
CA09 H1pdm	Significantly reduced	Significantly reduced	Significantly reduced	≤10 (53^d^)	Protection	6	0.63
OH10 H1γ	Significantly reduced	Not available	Significantly reduced	≤10 (109)	Protection	10	1.64
MN11 H1γ	Significantly reduced	Significantly reduced	Significantly reduced	≤20 (117)	Protection	11	2.10
IL08 H1α	Not significantly different	Significantly reduced	Significantly reduced	≤20 (240)	Partial protection	12	‐1.34

a Significance of outcomes was as measured and reported in the original references.

b HI GMT to the challenge and homologous viruses are shown.

c The vaccine was considered protective if it reduced macroscopic pneumonia and virus titers in nasal swabs and/or in lungs collected at necropsy. If the vaccine significantly reduced virus titers, but not lung lesions, it was considered partially protective.

d HI GMT to a heterologous γ‐cluster virus (A/Swine/OH/51145/2007 H1N1).

**Figure 5:**
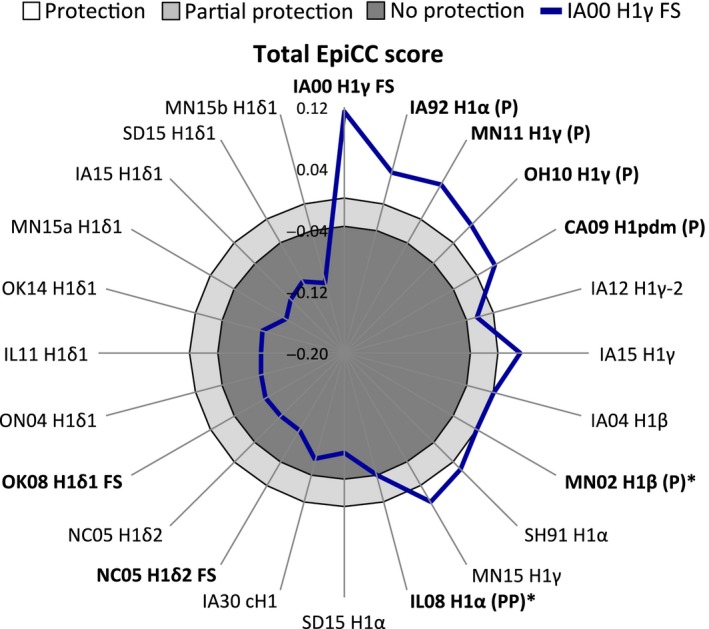
Definition of threshold for prediction of vaccine efficacy prediction. Total EpiCC scores (sum of class I and II EpiCC scores; blue line) for the comparison of H1γ FS and each viral strain are shown. The FS γ‐cluster vaccine strain was protective or partially protective against challenge with viruses annotated as (P) or (PP), respectively. The rest of the viruses were not tested as challenge strains. Protection and partial protection thresholds (black lines) defined 3 areas shown in white (protection; total EpiCC score above −0.001), light gray (partial protection), and dark gray (no protection). Viruses used to set the thresholds are marked with an asterisk (*). We hypothesize that FS would confer at least partial protection against challenge with viruses that had EpiCC scores outside the darker gray region

For the 6 vaccine efficacy studies considered in this analysis, FS conferred protection against challenge with 5 different H1N1 cluster viruses (Table 2). In 5 of the 6 studies, protection was conferred despite low levels of HI cross‐reactive antibodies (HI GMT to challenge virus ≤20) measured at the day of the challenge. For these studies, the threshold associated with protective efficacy was defined as the lowest total EpiCC score (EpiCC score of −0.001; MN02 H1β) comparing these 5 challenge strains with the vaccine strain (IA00 H1γ FS). This threshold defines the white area in Figure 5. For strains with EpiCC scores above the threshold, the scores of shared epitopes represented at least 67% (0.076) of the vaccine's baseline HA EpiCC score (0.114; Table 1), which may suggest that a field virus or a vaccine strain (or both) may have many unique epitopes, but as long as there is a sufficient level of shared epitopes relative to the baseline, an inactivated vaccine will be protective.

Additionally, the IA00 H1γ vaccine induced partial protection against challenge with IL08 H1α. This EpiCC score (−0.038) was considered a threshold associated with partial protection. This threshold separates the light gray from the dark gray area in Figure 5. IL08 H1α's shared epitopes score was 58.4% (0.066) of the vaccine's baseline EpiCC score. EpiCC scores for the H1δ cluster viruses, IA30 cH1 and SD15 H1α strains were below the partial protection threshold in the dark gray area.

Based on the association between total EpiCC scores of HA and vaccine efficacy, we speculate that immunization with the IA00 H1γ FS vaccine strain could confer protection against challenge with viruses with scores above −0.001 (white area in Figure 5) and partial protection for challenge‐vaccine EpiCC scores between ‐0.001 and −0.038 (light gray area in Figure 5), assuming minimal variation in internal antigens. Although specific vaccine efficacy data were not available for heterologous challenges performed with viruses for which EpiCC scores fall in the dark gray area of Figure 5, we would hypothesize that the vaccine might not be protective against these strains. Important differences between strain identity and EpiCC scores can be identified; for example, the nucleotide sequence of IA12 H1γ‐2 virus HA is highly identical to IA00 H1γ FS (93.36%); however, its total EpiCC score (−0.023) is below the threshold associated with protection, but above the threshold for partial protection. The low total EpiCC score is driven by a low score for shared epitopes (0.069; 60.2%) and high score for unique epitopes (0.092; Table 1). Nevertheless, shared epitopes between IA00 H1γ FS and IA12 H1γ‐2 virus HA might still contribute to a certain level of protection.

## 4 DISCUSSION

EpiCC is a method for assessing the relationship between field and vaccine strains of pathogens using predicted T‐cell epitope content as a metric for comparison; here it is applied to IAV and vaccination/challenge studies performed in swine. As compared to standard methods for estimating vaccine efficacy, such as determining whether immunization induces cross‐reactive antibodies to the HA proteins, or measuring genetic differences by sequence similarity, EpiCC characterizes the differences based on portions of the virus that the immune system processes and presents to T cells that drive potentially protective responses.

In our first test of the EpiCC scoring system, we compared the T‐cell epitope content of 20 HA sequences from different H1 clusters present in the North American swine population to that of 3 HA sequences from H1 viruses contained in a commercial swine IAV inactivated vaccine. To evaluate whether T‐cell epitope relatedness between vaccine and non‐homologous challenge strains was associated with protection, we compared EpiCC scores with experimental outcomes of efficacy studies of FS H1N1γ vaccine virus where protection was induced *in the absence of high levels of cross‐reactive HA antibodies*. The results of the analysis, performed without foreknowledge of efficacy outcomes, showed a threshold of T‐cell epitope relatedness that explained protection.

We do not yet know whether the threshold score would apply to new strains or different vaccines. The thresholds described in this study were based on experimental data from only 6 vaccine efficacy studies against challenge with cH1N1‐lineage viruses. Additional efficacy studies would help to refine and validate the thresholds for prediction of protection and partial protection as well as lack of protection, and permit the evaluation of permutations of the EpiCC calculation. In this version of the EpiCC calculation we prioritized epitope content shared between sequences and penalized strain‐ and vaccine‐unique epitopes; this is based on an assumption that response to shared epitopes is protective while response to unique epitopes is not, which may not be entirely true. Further studies will be required to extend these findings to other IAV strains, to determine whether EpiCC scores can be used to define thresholds of vaccine efficacy for other important swine pathogens, and whether EpiCC could be used to select influenza strains for human vaccines.

We note that the set of MHC alleles used for the prediction of epitopes influenced the scores of shared and unique epitopes, and therefore the EpiCC scores, between vaccine strains and field viruses. To illustrate this point, baseline EpiCC scores calculated using binding predictions to SLA alleles were shown to be different from those determined using a set of supertype HLA alleles. EpiCC scores for SLA‐I alleles were significantly lower than those for HLA‐I alleles and SLA‐II‐based scores were higher than HLA‐II EpiCC scores. For this study, SLA allele selection was based on frequencies determined using low‐resolution haplotyping for a small number of pigs.^22^ The relevance of these differences using distinct sets of MHC alleles is unknown; however, the distribution of SLA alleles for the North American swine population has yet to be defined, and therefore, the EpiCC scores might be different using a more comprehensive set of alleles. Development of a high‐throughput SLA typing system paired with a systematic study of SLA diversity would improve the utility of the EpiCC analysis for swine populations not only for IAV, but also for other economically important pathogens affecting the swine industry. EpiCC scores were developed for populations of swine (and humans). Some vaccines may be more or less protective for a given individual, depending on the EpiCC scores for that individual's SLA (or HLA) alleles.

In this case study, we were unable to determine which component of the score (shared class II epitopes or shared class I epitopes, for example), is more important for predicting protection. Published information describing T cell–dependent (CMI) responses elicited by swine IAV vaccines is scarce and some studies have reported that CMI responses to inactivated vaccines can be limited in pigs.^36^ However, other studies showed that inactivated vaccines can prime the CD4 + CD8 +  (double‐positive) memory T‐cell subset.^9^, ^37^, ^38^ Porcine CD4 + CD8 +  T cells are MHC class II‐restricted memory cells that express perforin and mediate cytolytic activity against virus‐infected cells.^39^, ^40^ For the set of alleles used for epitope prediction in this analysis, we found higher scores for class II epitopes shared between vaccines and field virus HAs compared to those of class I. Should further studies determine that cross‐reactive class II epitopes are more relevant for protection with inactivated vaccines, a weighted EpiCC score that favors class II epitopes could be applied.

Based on these findings, EpiCC scores showing high levels of T‐cell epitope relatedness support the hypothesis that T‐cell responses are involved in protection against challenge that is observed in the absence of HI cross‐reactivity in experimental efficacy studies of the FS γ‐cluster vaccine virus. The IA00 H1γ FS vaccine virus is clearly genetically and antigenically (by HI titer) distinct from the challenge viruses used in the 6 experimental challenge studies for which efficacy data were available. However, under the conditions of these experimental studies, vaccination with FS provided protection or partial protection against MN11 H1γ, IA92 H1α, OH10 H1γ, and CA09 H1pdm with HI titers lower than 1:40 (the cutoff generally considered predictive of protection by antibodies).^6^, ^9^, ^10^, ^11^ We observe that MN11 H1γ, IA92 H1α, and OH10 H1γ had the highest EpiCC scores among evaluated HA sequences when compared to IA00 H1γ FS; CA09 H1pdm had the sixth highest score. Among these H1N1 viruses, only IA92 H1α and CA09 H1pdm have different internal genes.^3^ IA92 H1α predates the emergence of TRIG, and CA09 H1pdm was classified as a swine‐origin IAV because internal and HA gene segments were genetically similar to those in the triple‐reassortant viruses circulating in North American swine.^41^ Some differences in strain‐specific T‐cell epitope content of internal proteins should be expected. Notwithstanding these potential differences in the internal genes, the IA00 H1γ FS vaccine was protective against challenge with both strains.^6^, ^9^ This result may suggest that a certain level of shared T‐cell epitopes could be associated with protection, despite the presence of unique epitopes. Antibodies to other surface antigens, such as NA, may have also played a role in protection.

The role of T‐cell epitopes in the highly variable external IAV protein, HA in protection against influenza in pigs is not yet known; however, human studies showed that vaccination with a monovalent subunit CA09 H1pdm vaccine elicited robust HA‐specific CD4 T‐cell responses dominated by memory CD4 T cells specific for peptides shared between the seasonal and pandemic strain.^42^ Researchers also demonstrated that expansion of CD4 T cells specific for peptide epitopes within HA, but not NP, correlated with neutralizing antibody response.^42^ These results support the notion that a greater degree of CD4 T‐cell cross‐reactivity (and higher class II EpiCC scores) may be associated with improved antibody response.

T cell–mediated responses directed to the conserved internal proteins of influenza viruses may have contributed to protection, as epitopes are more highly cross‐conserved than the epitopes found in HA.^43^, ^44^ In pigs, 2 evolutionary lineages (H1pdm09 and TRIG) dominate the selection of internal genes in the circulating influenza viruses, leading to a high degree of conservation in the internal genes.^3^, ^23^, ^24^ We have previously identified highly conserved SLA‐restricted epitopes in the NA and M proteins that are found in different IAV subtypes.^22^ Other groups have also reported conserved SLA class I‐restricted epitopes in NA.^45^, ^46^ This EpiCC analysis focuses on HA (while disregarding conserved internal and other external genes), as much of the antigenic variability between IAV strains circulating in swine (and differences in protection by vaccine strains) is localized to the HA surface antigen. Moreover, the predominant antibody response induced by inactivated vaccines is driven by antibodies to the HA protein. Therefore, these results are most relevant to inactivated vaccines, whole virus and HA subunit vaccines, and vectored vaccines containing the HA protein. Future studies will compare the utility of including other surface antigens (such as NA and M2) in the EpiCC score to determine whether thresholds revealed by HA‐specific T‐cell epitope relatedness can be further refined.

Genetic sequence comparison of HA is also used for predicting potential cross‐protective efficacy of vaccines. The relationship between EpiCC scores and HA amino acid sequence identity was nonlinear. At approximately 40% identity, epitope‐based relatedness was similar to the EpiCC score of a random amino acid sequence of the same length. And while changes in amino acids affecting T‐cell epitopes can have a significant impact on the immunogenicity of an antigen,^47^, ^48^ their effect on whole antigen sequence similarity may be minimal. To illustrate this point, we found viruses that had low EpiCC scores despite having high sequence identity. For example, when IA00 H1γ FS is compared to IA12 H1γ‐2, the nucleotide identity is 93.36% and the EpiCC score is above the threshold set for partial protection, but below the threshold for protection. Although H1γ‐2 cluster viruses were infrequently detected in the US swine population, characterization of H1γ‐2 viruses demonstrated divergent antigenic properties with viruses within the same clade and viruses from contemporary swine H1 clusters as well as commercial vaccines, suggesting a potential risk of vaccine failure against some H1γ‐2 viruses.^3^ On the contrary, EpiCC analysis suggests that the H1γ cluster vaccine virus may induce at least partial protection against IA12 H1γ‐2 virus.

In conclusion, we developed the EpiCC algorithm to assess the immunological relatedness between vaccines and emerging pathogen antigens based on their predicted T‐cell epitope content. Using EpiCC in a case study of swine influenza viruses, we found that vaccine protection conferred by the FS IA00 H1 γ‐cluster, in the absence of HI cross‐reactive antibodies, might be explained by predicted T‐cell epitope content relatedness between challenge and vaccine viruses. Based on these results, we proposed EpiCC score thresholds for prediction of full and partial protection. EpiCC scores were dependent on a set of swine MHC alleles used for the predictions of epitopes; thus, future EpiCC scores for these sequences may differ from the scores reported here. As information about SLA prevalence in North American swine populations becomes available, the impact of MHC allele selection on EpiCC scores will be assessed further.

This study provides preliminary evidence that EpiCC may complement current methods (HI cross‐reactivity and phylogenetic analysis) for selecting the best‐matched vaccine virus for immunization against emerging swine IAVs. Additional data from vaccine efficacy studies in swine and other species will be useful to validate and optimize these thresholds and extend the usefulness of EpiCC to other human and swine pathogens and available (or future) vaccines.

## CONFLICT OF INTERESTS

Funding for this project was provided by University of Rhode Island and Zoetis Inc. VRG was employed by Zoetis Inc. at the time this study was conducted. ADG and WDM are senior officers and majority shareholders at EpiVax, Inc., a privately owned immunoinformatics and vaccine design company located in Providence, RI, USA. LM and FET are employees at EpiVax, in which LM holds stock options. ADG, WDM, LM, and FET acknowledge that there is a potential conflict of interest related to their relationship with EpiVax and attest that the work contained in this research report is free of any bias that might be associated with the commercial goals of the company. And while Zoetis scientists participated in the analysis of the results and provided vaccine/challenge outcomes information, the EpiCC analysis was performed independently by AHG, ADG, and LM, limiting the potential for commercial bias.
