# Supplementary material for: T‐cell epitope content comparison (EpiCC) of swine H1 influenza A virus hemagglutinin
Source: Influenza Other Respir Viruses. 2017 Nov 28;11(6):531–42. doi: 10.1111/irv.12513 (PMC5705686; doi:10.1111/irv.12513)
Supplement: Supplementary file 1 [file IRV-11-531-s001.docx]

**Supplemental material**


**Supplemental Table 1. EpiCC scores between HA sequences of IA00 H1γ FS vaccine virus and field viruses.**

|  | **Score** | | | | **Nucleotide identity, %** | **Amino acid identity, %** |
| --- | --- | --- | --- | --- | --- | --- |
| **Strain^a^** | **Shared (%)^b^** | **Vaccine unique** | **Strain unique** | **Total EpiCC^c^** |  |  |
| IA00 H1γ FS | 0.114 (100) | 0.000 | 0.000 | 0.114 | 100 | 100 |
| MN11 H1γ (P) | 0.094 (83.0) | 0.022 | 0.023 | 0.050 | 94.18 | 95.05 |
| IA92 H1α (P) | 0.090 (79.4) | 0.027 | 0.023 | 0.040 | 94.94 | 95.58 |
| OH10 H1γ (P) | 0.088 (77.3) | 0.028 | 0.028 | 0.033 | 93.83 | 94.52 |
| IA15 H1γ | 0.087 (76.2) | 0.030 | 0.029 | 0.028 | 93.12 | 93.82 |
| CA09 H1pdm (P) | 0.084 (73.9) | 0.032 | 0.027 | 0.025 | 93.65 | 93.82 |
| MN15 H1γ | 0.084 (74.2) | 0.034 | 0.030 | 0.021 | 92.11 | 93.45 |
| SH91 H1α | 0.082 (72.0) | 0.035 | 0.035 | 0.011 | 92.42 | 93.11 |
| IA04 H1β | 0.076 (67.0) | 0.040 | 0.036 | 0.000 | 92.77 | 92.23 |
| MN02 H1β (P)* | 0.076 (67.0) | 0.041 | 0.037 | -0.001 | 92.71 | 92.05 |
| IA12 H1γ-2 | 0.069 (60.2) | 0.048 | 0.043 | -0.023 | 93.36 | 90.46 |
| IL08 H1α (PP)* | 0.066 (58.4) | 0.051 | 0.053 | -0.038 | 88.42 | 89.93 |
| IA30 cH1 | 0.059 (51.8) | 0.060 | 0.059 | -0.059 | 84.30 | 87.10 |
| SD15 H1α | 0.055 (48.4) | 0.062 | 0.063 | -0.070 | 86.89 | 87.63 |
| OK08 H1δ1 FS | 0.052 (45.3) | 0.066 | 0.069 | -0.083 | 75.97 | 78.94 |
| NC05 H1δ2 FS | 0.051 (44.9) | 0.066 | 0.070 | -0.086 | 76.50 | 80.53 |
| NC05 H1δ2 | 0.051 (44.9) | 0.066 | 0.071 | -0.086 | 76.38 | 80.71 |
| ON04 H1δ1 | 0.051 (44.6) | 0.068 | 0.075 | -0.092 | 75.97 | 79.12 |
| IL11 H1δ1 | 0.048 (42.2) | 0.070 | 0.072 | -0.094 | 75.85 | 78.05 |
| OK14 H1δ1 | 0.050 (44.0) | 0.068 | 0.076 | -0.094 | 75.74 | 78.94 |
| SD15 H1δ1 | 0.047 (41.4) | 0.071 | 0.072 | -0.096 | 75.62 | 77.88 |
| IA15 H1δ1 | 0.048 (41.9) | 0.071 | 0.081 | -0.104 | 75.68 | 78.94 |
| MN15b H1δ1 | 0.046 (40.7) | 0.072 | 0.084 | -0.110 | 75.56 | 78.58 |
| MN15a H1δ1 | 0.043 (37.6) | 0.077 | 0.082 | -0.116 | 75.68 | 78.41 |

^a^FS γ-cluster vaccine strain was protective or partially protective against challenge with viruses annotated as (P) or (PP), respectively. Viruses used to set the thresholds are marked with an asterisk (*).

^b^Ratio of the score of shared epitopes relative to the baseline EpiCC scores expressed as percentage.

^c^Sum of class I and II EpiCC scores.

Table is sorted by total EpiCC score. Strains below the total EpiCC score threshold associated with partial protection are shown in gray.

**Supplemental Figure 1. Illustration of T cell epitope content comparison (EpiCC) score calculation.**

**
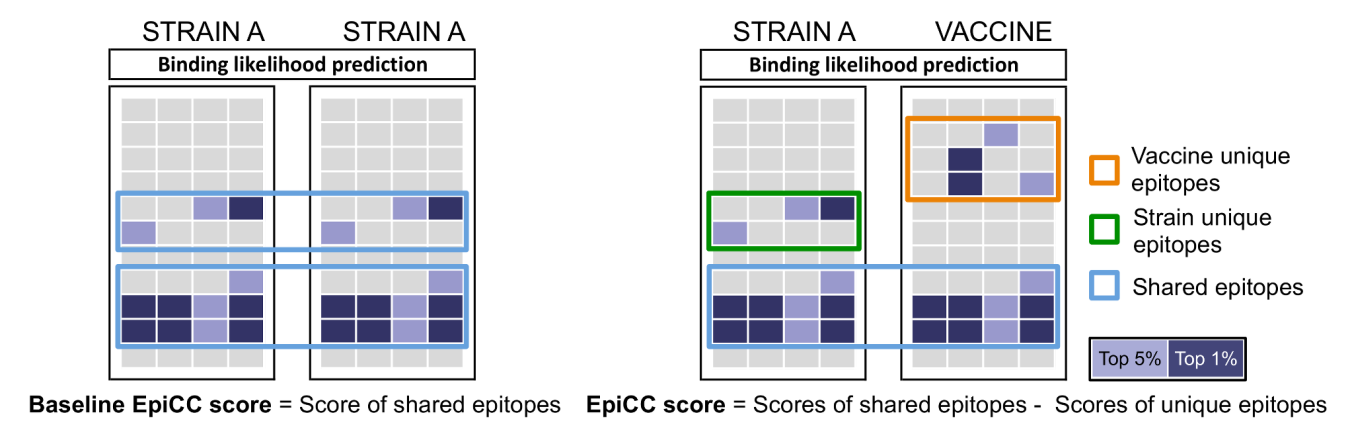
**

Strain A and Vaccine were screened for binding likelihood to a set of four MHC alleles; 9-mers (rows) predicted to bind to specific MHC alleles (columns) are shown in light (top 5%) or dark (top 1%) blue. The comparison of the epitope content of Strain A to itself determines the baseline EpiCC score (left). For the comparison between Strain A and Vaccine, scores of shared and unique epitopes are considered when calculating the EpiCC score (right).

**Supplemental Figure 2. Comparison of HA baseline EpiCC score by set of MHC alleles.**

**
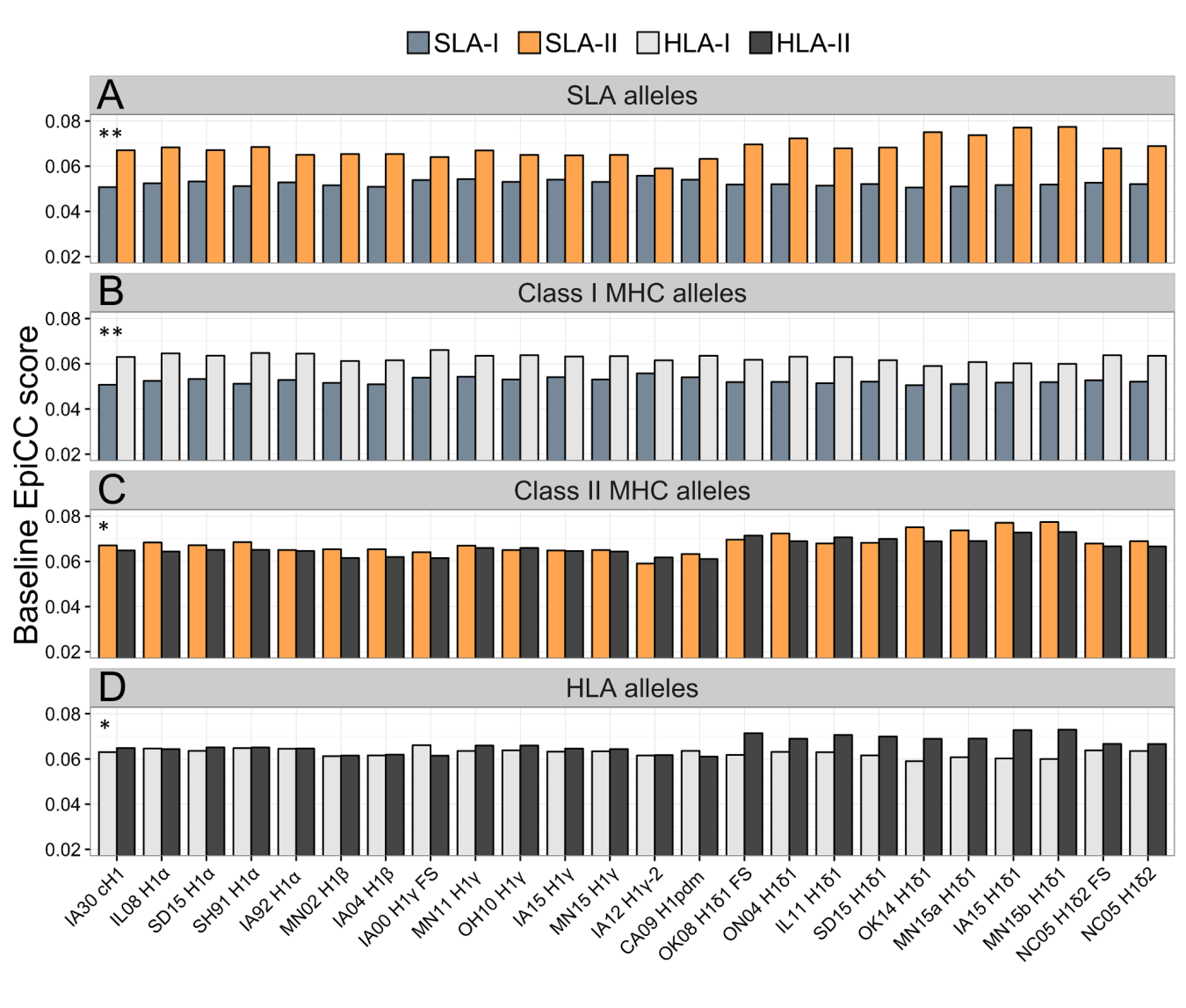
**

Baseline EpiCC score of HA sequences of each virus (***E***(***s***,***s***)***_A_***) was determined using predictions for swine and human class I and II MHC alleles (SLA-I: 8 alleles, SLA-II: 5 alleles, HLA-I: 6 alleles, and HLA-II: 8 alleles). HA baseline EpiCC score comparisons between A) SLA-I and SLA-II, B) SLA-I and HLA-I, C) SLA-II and HLA-II, and D) HLA-I and HLA-II, are shown. P-values of comparisons were calculated using one-tailed Wilcoxon matched-pairs signed rank test (**p<0.001, *p<0.05).

**Supplemental Figure 3. Comparison of scores of shared and unique epitopes across strains.**

**
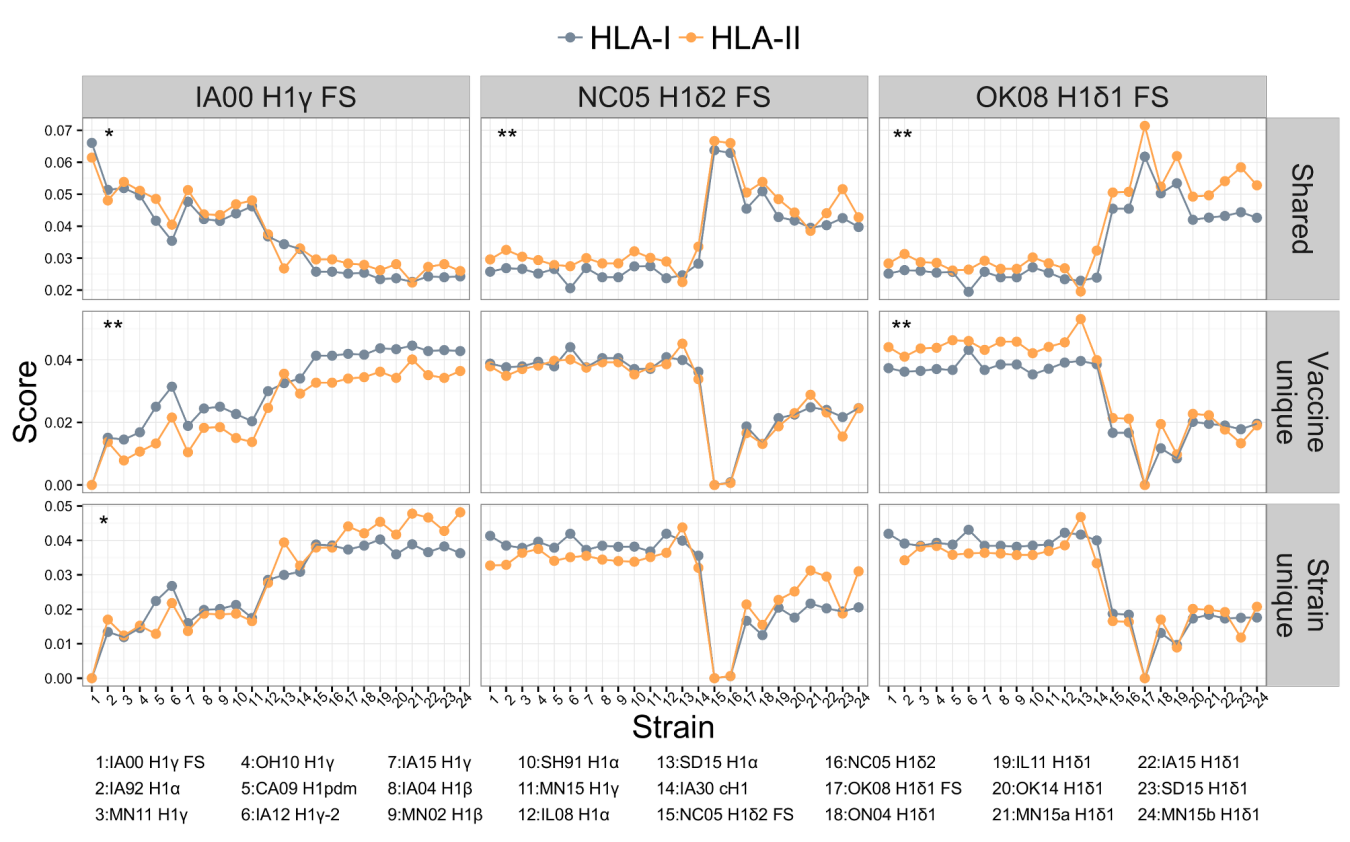
**

Scores of shared, vaccine unique and strain unique HLA class I and II epitopes were determined for the comparison of HA sequences from vaccine viruses and field (challenge) strains. Note that y-axes show different scales. Solid connecting lines are included only for visualization purposes. P-values of comparisons were calculated using one-tailed Wilcoxon matched-pairs signed rank test (**p<0.001, *p<0.01). HA vaccine sequences had higher scores for shared epitopes with strains belonging to the same H1 cluster or the same HA lineage. Scores of class II shared epitopes were significantly higher than those of class I. Class II scores of unique epitopes were also higher for OK08 H1δ1 FS (vaccine unique) and NC05 H1δ2 FS (strain unique). In contrast, class I scores of IA00 H1γ FS vaccine unique epitopes were higher than those of class II, which is explained by the higher baseline EpiCC score of the vaccine. Viruses are sorted by nucleotide identity relative to H1γ FS. Strain numbers on the x-axis are described in detail in the legend below.

**Supplemental Figure 4. Relationship between EpiCC scores and nucleotide identity.**


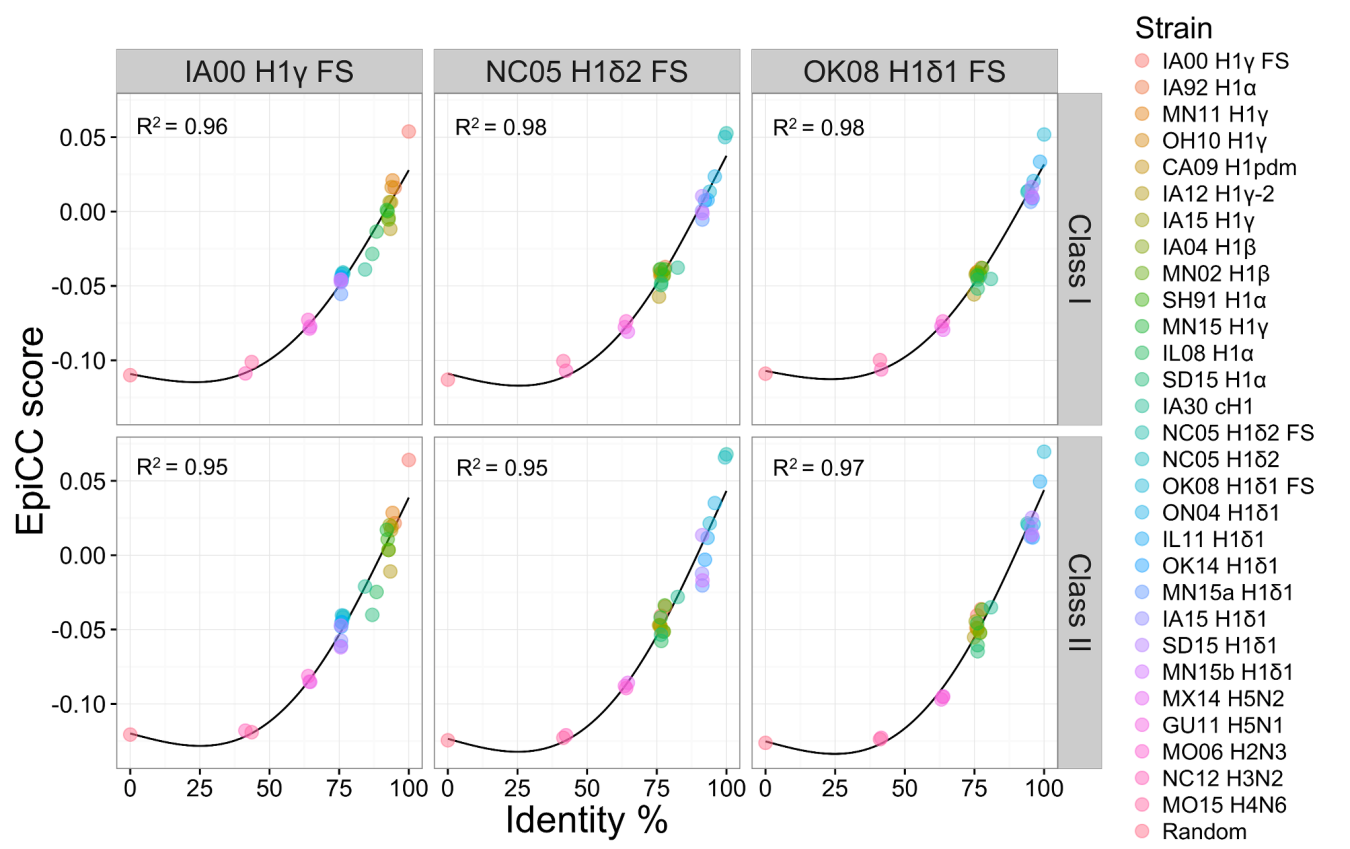


The second order polynomial relationship between class I (top), II (bottom) EpiCC scores, and nucleotide identity for each FS virus are shown. R^2^ of regression models are shown. H2N3, H5N1, H5N2, H3N2, H4N6 and a random sequence were included in this analysis to represent the lower end of the identity range. Class I and II EpiCC scores correlated with identity between HA nucleotide sequences of vaccines and viral strains (r=0.86 – 0.88). However, their relationship was nonlinear; instead, it was second order polynomial (R^2^=0.94 – 0.98). EpiCC score was close to the lowest at approximately 50% identity.
